# Efficacy and safety of two pegfilgrastim biosimilars: Tripegfilgrastim and pegteograstim

**DOI:** 10.1002/cam4.3261

**Published:** 2020-07-07

**Authors:** Ka‐Won Kang, Byung‐Hyun Lee, Min Ji Jeon, Eun Sang Yu, Dae Sik Kim, Se Ryeon Lee, Hwa Jung Sung, Chul Won Choi, Yong Park, Byung Soo Kim

**Affiliations:** ^1^ Division of Hematology‐Oncology Department of Internal Medicine Korea University College of Medicine Seoul South Korea

**Keywords:** biosimilar, febrile neutropenia, neutropenia, pegylated granulocyte‐colony stimulating agent

## Abstract

Our aim was to compare the efficacy and safety of two recently developed biosimilars of pegfilgrastim, a pegylated form of the recombinant human granulocyte‐colony stimulating factor (G‐CSF) analog filgrastim with those of the reference pegfilgrastim. We retrospectively analyzed data from patients diagnosed with diffuse large B‐cell lymphoma (DLBCL) who were treated with first‐line R‐CHOP chemotherapy and received pegylated G‐CSF for primary prophylaxis. The following pegylated G‐CSFs were analyzed in this study: reference pegfilgrastim (Neulasta^®^) and two of its biosimilars (tripegfilgrastim; Dulastin^®^ and pegteograstim; Neulapeg^®^). In total, 296 patients were enrolled. The number of patients with at least one episode of neutropenia during R‐CHOP chemotherapy was the lowest in the reference cohort (pegfilgrastim: 127 of 193 patients, 65.8%; tripegfilgrastim: 64 of 69 patients, 92.8%; pegteograstim: 28 of 34 patients, 82.4%, *P* < .001). The number of patients with at least one episode of febrile neutropenia was also lowest in the reference cohort (pegfilgrastim: 67 of 193 patients, 34.7%; tripegfilgrastim: 38 of 69 patients, 55.1%; pegteograstim: 16 of 34 patients, 47.1%, *P* = .009). There were no differences in the duration of neutropenia and febrile neutropenia or treatment outcomes (rate of complete response or relapse and survival). There were no reports of grade 3 or higher adverse events requiring discontinuation of prophylactic pegylated G‐CSF in any group. The safety of the pegfilgrastim biosimilars for prophylactic purposes was comparable to that of the reference pegfilgrastim; however, in terms of their efficacy, the incidence of neutropenia and febrile neutropenia tended to be higher than that when using pegfilgrastim. The clinical relevance of these results in the biosimilar cohorts should be explored.

## Graphical abstract

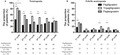

## 1 INTRODUCTION

Neutropenia and febrile neutropenia are serious complications of chemotherapy that may lead to treatment‐related mortality or affect treatment outcomes owing to the need to reduce the intensity of treatment or delay treatment.^1^ These issues are more pronounced in patients requiring high‐intensity chemotherapy for curative purposes, such as patients with hematologic malignancy. One of the advances in supportive care is the use of granulocyte‐colony stimulating factor (G‐CSF) to resolve these problems.^2^, ^3^

G‐CSF is one of the various extracellular stimuli that mediate blood cell production.^4^ It has been shown to play an important role in the survival and differentiation of neutrophil granulocytes and their progenitors,^5^, ^6^ increase in response to bacterial infection and the cell‐mediated immune response for emergency granulocyte production,^6^, ^7^, ^8^ and enhance the effector functions of mature neutrophils.^9^, ^10^ Consequently, G‐CSF is used for prophylaxis of neutropenia and/or febrile neutropenia and as one of the treatments for febrile neutropenia during chemotherapy.^3^, ^11^, ^12^

Pegylated G‐CSF, created by the covalent attachment of a polyethylene glycol moiety to G‐CSF, could be a better alternative to the conventional G‐CSF formulation because of its longer half‐life and sustained duration of action.^13^ A single dose of pegylated G‐CSF every 3‐4 weeks is as effective as daily injections of the conventional G‐CSF for stimulation of the neutrophil response, with no notable differences in toxicity.^14^ Pegylated G‐CSF is preferred for patients who require prophylactic G‐CSF; however, the cost of pegylated G‐CSF is high. Recently, various biosimilars of pegylated G‐CSFs have been used to overcome the cost limitations.

Even if the biosimilars of pegylated G‐CSFs are cost‐effective, the most important factors are their efficacy and safety. According to studies performed to date, there are no clinically meaningful differences in efficacy and safety between pegfilgrastim (the reference pegylated G‐CSF) and its biosimilars; however, most studies have been conducted in healthy volunteers or patients with solid cancers.^15^, ^16^, ^17^, ^18^, ^19^ No study has compared pegfilgrastim biosimilars with pegfilgrastim in patients with hematologic malignancy, who usually have an intermediate or high risk of febrile neutropenia.^3^, ^11^, ^12^

This study was a non‐interventional cohort study and aimed to confirm the efficacy and safety of two pegfilgrastim biosimilars in comparison with those of the reference pegfilgrastim in patients with clinically equivalent conditions. The study subjects were patients who were diagnosed with diffuse large B‐cell lymphoma (DLBCL), the most common hematologic malignancy, and treated with R‐CHOP chemotherapy as the first‐line therapy.

## 2 METHODS

### 2.1 Study design and patients

This was a non‐interventional comparative cohort study in which we retrospectively analyzed the data of patients who were consecutively enrolled in the Lymphoma Registry from January 2014 to February 2020. The study was approved by the institutional review board, and all data were fully anonymized.

Patients who met the following inclusion criteria were selected: (a) histologic diagnosis of DLBCL; (b) received R‐CHOP chemotherapy as the first‐line therapy; and (b) received pegylated G‐CSF as the primary prophylaxis. If a patient used intravenous methotrexate for therapeutic or prophylactic purposes, they were excluded from the analysis. However, if a patient used intrathecal methotrexate for prophylactic purposes, the patient was included. The following pegylated G‐CSFs were analyzed in this study: reference pegfilgrastim (Neulasta^®^, from 2014 until the present year [February 2020]), tripegfilgrastim (Dulastin^®^, from 2015 until the present year), and pegteograstim (Neulapeg^®^, from 2017 until the present year).

R‐CHOP chemotherapy consisting of 375 mg/m^2^ intravenous rituximab, 750 mg/m^2^ cyclophosphamide, 50 mg/m^2^ adriamycin, and 1.4 mg/m^2^ vincristine (maximum dose: 2 mg) on day 1, and 100 mg/day oral prednisone on days 1‐5, was administered every 21 days for a total of six to eight cycles. Pegylated G‐CSFs (pegfilgrastim, tripegfilgrastim, and pegteograstim) were dosed as a 6 mg single injection on day 2, 24 hours after the intravenous chemotherapy was completed. Levofloxacin (500 mg, once daily from days 1 through 10 of each cycle) and sulfamethoxazole/trimethoprim (400 mg/80 mg each, twice daily from days 1 through 7 of each cycle) were used as concomitant prophylaxis for febrile neutropenia. To be included, patients had to have completed at least one cycle of R‐CHOP. At the time of analysis, all patients had finished first‐line therapy.

The surveillance protocol for adverse events during R‐CHOP chemotherapy in our center was as follows. The first cycle of R‐CHOP was performed with most patients hospitalized, and a complete blood count (CBC) was conducted daily from day 7 or 8 to check the absolute neutrophil count (ANC). After identification of the day on which the lowest ANC occurred during the first cycle, in the second cycle, we performed a CBC for 1 day or 2 days before that day. If the patient's ANC decreased to below 1000/µL, the CBC was performed daily until the ANC recovered to above 1000/µL; if the patient's ANC did not fall to below 1000/µL, the CBC was checked every 1‐3 days. The follow‐up CBC examination was performed for outpatients or inpatients as required by the patient's condition.

### 2.2 Clinical endpoints

The primary endpoint was the proportion of patients that experienced neutropenia or febrile neutropenia during chemotherapy and the duration of these conditions. Neutropenia was defined as an ANC of less than 1000/µL, and the duration of neutropenia was calculated as the median number of days from the first day of neutropenia to the time at which ANC > 1000/L was achieved. Febrile neutropenia was defined by the following conditions: (a) a temperature of >38.3°C or > 38.0°C sustained for 1 hour; (b) ANC < 500/µL or ANC < 1000/µL, predicted to decline to ≤ 500/µL over the next 48 hours. The duration of febrile neutropenia was calculated as the median number of days from the first day the definition of febrile neutropenia was met to the time at which ANC recovered to > 1000/µL and the antibiotic treatment for febrile neutropenia was completed.

Secondary endpoints included the number of chemotherapy adjustments (schedule delay, dose reduction, early discontinuation, and dose escalation) related to neutropenia or febrile neutropenia, response evaluation after completion of first‐line R‐CHOP, the rate of relapse after achieving complete response (CR), progression‐free survival (PFS), overall survival (OS), infection incidence, and the incidence of adverse events higher than grade 3 causing discontinuation of the pegylated G‐CSF. The response evaluation and relapse were assessed by the Lugano classification.^20^ In patients who had achieved CR, the duration of PFS was defined as the time from the date of CR to the date of relapse or death. OS was defined as the time from the date of diagnosis to the date of death. Adverse events higher than grade 3 were assessed by using the National Cancer Institute's Common Toxicity Criteria for Adverse Events (version 5.0).

### 2.3 Statistical analysis

Baseline characteristics were compared using the Kruskal–Wallis H test or Chi‐squared test, as appropriate, and a post hoc analysis with Bonferroni correction was performed if needed. The proportion of patients that experienced neutropenia or febrile neutropenia during chemotherapy, the rate of response, and the rate of relapse were compared by using a Chi‐squared test, and the duration of neutropenia or febrile neutropenia was compared using a Kruskal–Wallis H test with post hoc Bonferroni correction. PFS and OS were calculated using a Kaplan–Meier survival analysis and were compared using the log‐rank test. The IBM Statistical Package for Social Sciences (SPSS) version 21.0 (IBM Corp., Armonk, NY, USA) was used for data analysis. A p‐value of < 0.05 was considered statistically significant.

## 3 RESULTS

### 3.1 Patient characteristics

In total, 296 patients were enrolled in this study. The reference pegfilgrastim cohort included 193 patients, and the tripegfilgrastim cohort and pegteograstim cohort included 69 and 34 patients, respectively. The baseline characteristics at diagnosis for each group are summarized in Table 1. In the reference pegfilgrastim cohort, more patients had the unclassified subtype than in other cohorts, and there was a relatively low proportion of non‐germinal center types (*P* < .001). Apart from this, there were no significantly different characteristics among the groups, including age, sex, Eastern Cooperative Oncology Group performance scores, stage, bone marrow involvement, and relative dose intensity of the first cycle.

**TABLE 1:** Baseline characteristics at diagnosis

Baseline characteristics at diagnosis	Pegfilgrastim (n = 193)	Tripegfilgrastim (n = 69)	Pegteograstim (n = 34)	*P*
Median age, years (range)	63 (26‐85)	65 (27‐92)	63 (29‐84)	.719
<60 ys, n (%)	78 (40.4)	27 (39.1)	12 (35.3)	.874
≥60 y, n (%)	115 (59.6)	42 (60.9)	22 (20.6)	
Male:Female ratio	1.54	0.97	1.43	.262
ECOG, n (%)				.060
0‐1	191 (98.9)	68 (98.6)	32 (94.1)	
≥2	2 (1.0)	1 (1.4)	2 (5.8)	
DLBCL, subtype, n (%)				<.001
Non‐GCT	87 (45.1)	46 (66.7)	23 (67.6)	
GCT	48 (24.9)	18 (26.1)	8 (23.5)	
Unknown	58 (30.1)	5 (7.2)	3 (8.8)	
Stage, n (%)				.596
I‐II	104 (53.9)	37 (53.6)	15 (44.1)	
III‐IV	89 (46.1)	32 (46.4)	19 (55.9)	
BM involvement, n (%)	13 (6.7)	5 (7.2)	3 (8.8)	.919
Relative dose intensity of 1st cycle (%)				.974
Median (range)	100 (50‐100)	100 (50‐100)	100 (50‐100)	
Mean (SD)	87.95 (15.29)	89.64 (14.56)	88.97 (15.56)	

Abbreviations: BM, bone marrow; DLBCL, diffuse large B‐cell lymphoma; ECOG, Eastern Cooperative Oncology Group performance; GCT, germinal center type; SD, standard deviation.

### 3.2 Comparison of effectiveness endpoints

The median number of cycles per patient was six (range: 2‐6) in the reference pegfilgrastim cohort, six (range: 1‐6) in the tripegfilgrastim cohort, and six (range: 3‐6) in the pegteograstim cohort. The number of patients with at least one episode of neutropenia during first‐line R‐CHOP chemotherapy was lowest in the reference pegfilgrastim cohort (reference pegfilgrastim: 127 of 193 patients, 65.8%; tripegfilgrastim cohort: 64 of 69 patients, 92.8%; pegteograstim: 28 of 34 patients, 82.4%, *P* < .001). The number of patients with at least one episode of febrile neutropenia during first‐line R‐CHOP chemotherapy was also lowest in the reference pegfilgrastim cohort (reference pegfilgrastim: 67 of 193 patients, 34.7%; tripegfilgrastim cohort: 38 of 69 patients, 55.1%; pegteograstim: 16 of 34 patients, 47.1%, *P* = .009) (Table 2).

**TABLE 2:** Comparison of effectiveness endpoints

Effectiveness endpoints	Pegfilgrastim (n = 193)	Tripegfilgrastim (n = 69)	Pegteograstim (n = 34)	*P*
Number of patients with at least one N episode during CTx, n (%)	127 (65.8)	64 (92.8)	28 (82.4)	<.001
Number of patients with at least one FN episode during CTx, n (%)	67 (34.7)	38 (55.1)	16 (47.1)	.009
Number of chemotherapy adjustments due to N or FN				—
Schedule delay	1 (0.6)	1 (1.5)	0 (0)	
Dose reduction	11 (8.4)	1 (1.5)	1 (1.5)	
Early discontinuation	6 (3.4)	7 (10.6)	3 (1.9)	
Dose escalation	3 (1.7)	3 (4.5)	4 (12.1)	

Abbreviations: N, neutropenia; CTx, chemotherapy; FN, febrile neutropenia.

**FIGURE 1:**
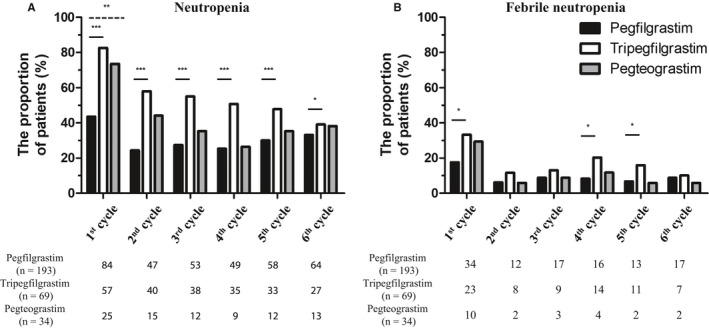
Incidence of neutropenia and febrile neutropenia during first‐line R‐CHOP chemotherapy. ***P < .001; **P < .01; and *P < .05 after Bonferroni correction

Over all six cycles, the reference pegfilgrastim cohort showed lower overall neutropenia incidence during six cycles of R‐CHOP compared to the biosimilar cohorts (Figure 1A). The tripegfilgrastim cohort showed a significantly higher incidence of neutropenia than the reference pegfilgrastim cohort in all six cycles (*P* < .001, <.001, <.001, <.001, <.001, and .042 after Bonferroni correction, respectively). In the case of the pegteograstim cohort, only the first cycle showed a significantly higher incidence of neutropenia than the reference pegfilgrastim cohort (*P* = .003 after Bonferroni correction). Febrile neutropenia was shown to have a similar overall incidence except for the first cycle (Figure 1B). The tripegfilgrastim cohort had a significantly higher incidence of febrile neutropenia than the reference pegfilgrastim cohort in the 1st, 4th, and 5th cycles (*P* = .03, .012, and .018 after Bonferroni correction, respectively). However, there were no statistically significant differences in the pegteograstim cohort compared with the reference pegfilgrastim cohort.

**FIGURE 2:**
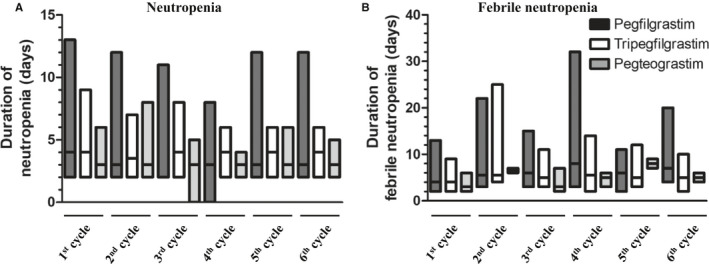
Duration of neutropenia and febrile neutropenia during first‐line R‐CHOP chemotherapy. The boundaries of the box indicate the minimum and maximum, and the line within the box marks the median

When neutropenia and febrile neutropenia occurred during each cycle, the duration of each did not differ among the cohorts (Figure 2A,B). The adjustments of chemotherapy owing to neutropenia or febrile neutropenia are summarized in Table 2. There was no difference in the frequency of the situations affecting the progress of treatment, such as delayed treatment, dose reduction of chemotherapy, and early discontinuation of treatment because of neutropenia or febrile neutropenia.

### 3.3 Effect on treatment outcomes

**FIGURE 3:**
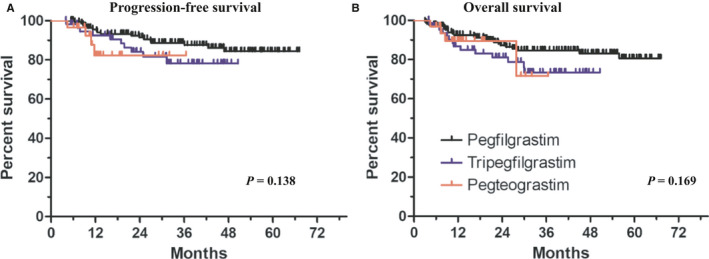
Progression‐free survival and overall survival according to the type of the pegylated G‐CSF treatment

The CR rates after completing first‐line R‐CHOP chemotherapy were 80.3% (155 of 193 patients), 82.6% (57 of 69 patients), and 91.2% (31 of 34 patients) in the reference pegfilgrastim, tripegfilgrastim, and pegteograstim cohorts, respectively. The relapse rates in patients who completed first‐line R‐CHOP chemotherapy and achieved CR according to the response evaluation criteria were 9.7% (15 of 193 patients), 14.3% (8 of 69 patients), and 10.0% (3 three 34 patients) in the reference pegfilgrastim, tripegfilgrastim, and pegteograstim cohorts, respectively. There were no statistically significant differences in the CR rates and relapse rates among the cohorts (Table 3). Similarly, the PFS and OS also did not vary significantly among the cohorts (Figure 3A,B).

**TABLE 3:** Effect on treatment outcomes

Effect on treatment outcomes	Pegfilgrastim (n = 193)	Tripegfilgrastim (n = 69)	Pegteograstim (n = 34)	*P*
Response evaluation after completion of first‐line chemotherapy, n (%)				.301
Complete response	155 (80.3)	57 (82.6)	31 (91.2)	
Partial response	22 (11.4)	4 (5.8)	2 (5.9)	
Progressive disease	15 (7.8)	6 (8.7)	1 (2.9)	
Relapse^a^	15 (9.7)	8 (14.3)	3 (10.0)	.674

^a^ All relapses were counted only in patients who completed first‐line R‐CHOP and achieved complete response according to the response evaluation criteria.

### 3.4 Documented infections and adverse events leading to discontinuation of the pegylated G‐CSF

Among patients with febrile neutropenia, documented infections occurred in 38.8% (26 of 67 patients in the reference pegfilgrastim cohort), 23.7% (9 of 38 patients in the tripegfilgrastim cohort), and 25.1% (4 of 16 patients in the pegteograstim cohort) (Table 4). When the documented infections were compared, there were no associations between specific bacteria and the treatment group. There were no reports of grade 3 or higher adverse events requiring discontinuation of the prophylactic pegylated G‐CSF in any group.

**TABLE 4:** Documented infections during first‐line R‐CHOP chemotherapy

Documented infections	Pegfilgrastim (n = 67)	Tripegfilgrastim (n = 38)	Pegteograstim (n = 16)
Number of microbiologically documented infections, n (%)	26 (38.8)	9 (23.7)	4 (25.1)
Blood	6 (9.0)	4 (10.5)	3 (18.8)
Acinetobacter species	1	━	━
Bacteroides species	1	━	━
Candida species	1	━	━
Clostridium species	━	1	━
Enterobacter species	1	━	━
Enterococcus species	━	1	2
Escherichia species	1	━	━
Staphylococcus species	1	2	1
Sputum	7 (10.4)	0 (0.0)	0 (0.0)
Acinetobacter species	1	━	━
Enterobacter species	2	━	━
Staphylococcus species	2	━	━
Streptococcus species	1	━	━
Pseudomonas species	1	━	━
Urine	13 (19.4)	5 (13.2)	1 (6.3)
Corynebacterium species	2	━	━
Enterococcus species	7	5	1
Escherichia species	3	━	━
Staphylococcus species	1	━	━

## 4 DISCUSSION

In this study, two pegfilgrastim biosimilars (tripegfilgrastim and pegteograstim) did not show severe toxicities and did not affect the outcome of treatment (rates of CR or relapse, and survival) during the first‐line R‐CHOP chemotherapy in patients newly diagnosed with DLBCL. However, in terms of the incidence of neutropenia and febrile neutropenia, the biosimilars showed lower efficacy compared with the reference pegfilgrastim, especially in the first cycle of R‐CHOP chemotherapy. When neutropenia and febrile neutropenia occurred during first‐line R‐CHOP chemotherapy, there were no differences in the duration of neutropenia and febrile neutropenia among the cohorts.

G‐CSF is a pleiotropic growth factor that stimulates the survival, proliferation, differentiation, and function of bone marrow precursor cells and mature neutrophils through its G‐CSF receptor (G‐CSFR). The activated G‐CSFR mediates downstream signal transduction pathways, such as Janus kinase (JAK)/signal transducer and activator of transcription (STAT), Src kinases, and phosphatidylinositol 3‐kinase (PI3K), resulting in the increased production of neutrophils in healthy and diseased states.^21^, ^22^ Pegylated G‐CSF is created by the conjugation of a polyethylene glycol moiety to the conventional G‐CSF, minimizing its renal clearance by glomerular filtration and making neutrophil‐mediated clearance the predominant route of elimination without interfering with its receptor‐binding domain.^13^ The difference between the reference pegfilgrastim and its two biosimilars is thought to be the balance between this adjustment of the elimination pathway and the binding affinity to G‐CSFR.

To date, published studies have shown no difference in efficacy and safety between reference pegfilgrastim and its biosimilars.^15^ However, there is not enough evidence to draw a definitive conclusion because the number of studies is small; most of these studies were conducted in patients with breast cancer, and the interpretation of efficacy was mainly focused on the duration of neutropenia.^23^, ^24^, ^25^ In this study, the biosimilars were associated with a higher incidence of neutropenia and febrile neutropenia than the reference pegfilgrastim, especially in the first cycle of R‐CHOP chemotherapy. Since the efficacy of pegylated G‐CSF could be affected by the function of its receptor‐binding domain and the avoidance of renal clearance, if these results are accurate, several hypotheses can be proposed. The first hypothesis is that the difference in receptor‐binding ability based on the drug may occur due to differences in the lymphoma itself or its specific treatment. Lymphoma, derived from lymphoid cell lines, may exhibit a stronger expression of G‐CSFR,^26^, ^27^, ^28^ resulting in dysregulated signaling pathways, including JAK/STAT, Src kinases, and PI3K, compared to solid cancers.^29^ In addition, the anti‐CD20 agents and high‐dose steroids used in the treatment of B‐cell lineage lymphoma have been associated with STAT,^30^, ^31^, ^32^ Src kinases,^33^, ^34^ and PI3K.^35^, ^36^ These molecular changes may have affected receptor‐binding ability and our ability to distinguish between the efficacy of the reference pegfilgrastim and the biosimilars. The second hypothesis is that the subjects of the present study are different from the subjects of previous studies.^23^, ^24^, ^25^ The median age of patients was 49.9‐53.4 years in previous studies; however, it was 63 years (range: 26‐92 years) in the present study. In the present study, at diagnosis, 1.4% of patients had neutropenia (ANC < 1000/µL) (3 of 193 patients, 1.6%; tripegfilgrastim: 0 of 69 patients, 0%; and pegteograstim: 1 of 33 patients, 2.9%), and 21.6% of patients showed less than 60 mL/min/1.73 m^2^ (estimated glomerular filtration rate calculated by the CKD‐EPI equation; pegfilgrastim: 38 of 193 patients, 19.7%; tripegfilgrastim: 16 of 69 patients, 23.1%; and pegteograstim: 10 of 33 patients, 29.4%). On the other hand, 7.1% of patients in the present study had bone marrow involvement with their lymphoma at the time of diagnosis (Table 1). However, since other studies did not provide information on ANC counts, kidney function, and bone marrow invasion of the underlying disease, direct comparison to the present study is not possible. In addition, although there was no statistical difference in the distribution of the above characteristics for each group in the present study, the possibility that the minute difference between the groups influenced the receptor‐binding ability and its renal clearance cannot be eliminated. However, the reasons why the biosimilars were associated with a higher incidence of neutropenia and febrile neutropenia than the reference pegfilgrastim cannot be determined by this study alone, and further studies are warranted.

This was a retrospective study, and because of the small number of patients in the analysis, there was a limit to the conclusions that could be drawn from the results. In addition, as only the biosimilars of pegylated G‐CSFs used in our center were compared and analyzed, our results cannot represent all biosimilars of pegylated G‐CSFs. Nevertheless, this study is, to the best of our knowledge, the first to compare the reference pegfilgrastim and its biosimilars in hematologic malignancy. We analyzed the efficacy and safety of the reference pegfilgrastim and its biosimilars in a uniform clinical setting by studying patients with DLBCL receiving first‐line R‐CHOP under an identical prophylactic and surveillance protocol for neutropenia and febrile neutropenia during chemotherapy. Therefore, this study can easily be applied in real clinical practice. As more evidence accumulates, it is predicted that the biosimilars will be put to use in clinical situations when indicated by evidence‐based analysis of the costs and benefits.

In conclusion, in patients with DLBCL receiving first‐line R‐CHOP chemotherapy, safety of the prophylactic use of pegfilgrastim biosimilars (tripegfilgrastim and pegteograstim) did not differ from that of the reference pegfilgrastim. However, in terms of efficacy, though the duration of neutropenia and febrile neutropenia did not differ, the actual incidence rate of neutropenia and febrile neutropenia was higher in patients treated with the biosimilars than in those treated with the reference pegfilgrastim. The clinical relevance of these results in the biosimilar cohorts warrants further evaluation.

## CONFLICT OF INTEREST

The authors declare that they have no competing interests.

## AUTHOR CONTRIBUTIONS

BSK and KWK designed the study. KWK analyzed the registry and summarized the results. KWK, BHL, MJJ, ESY, DSK, SRL, HJS, CWC, YP, and BSK performed patient management and maintained the registry. KWK wrote the manuscript. All authors approved the final version of the manuscript.

## Data Availability

The datasets during and/or analyzed during the current study are available from the corresponding author on reasonable request.

## References

[1] Cameron D . Management of chemotherapy‐associated febrile neutropenia. Br J Cancer. 2009;101(Suppl 1):S18‐S22. 10.1038/sj.bjc.6605272 PMC2752227 19756002

[2] Lyman GH , Poniewierski MS . A patient risk model of chemotherapy‐induced febrile neutropenia: lessons learned from the ANC study group. J Natl Comprehensive Cancer Network. 2017;15(12):1543‐1550. 10.6004/jnccn.2017.7038 29223991

[3] Smith TJ , Bohlke K , Lyman GH , et al. Recommendations for the use of WBC growth factors: American Society of Clinical Oncology clinical practice guideline update. J Clin Oncol. 2015;33(28):3199‐3212. 26169616 10.1200/JCO.2015.62.3488

[4] Basu S , Dunn A , Ward A . G‐CSF: function and modes of action (Review). Int J Mol Med. 2002;10(1):3‐10. 12060844

[5] Avalos BR . Molecular analysis of the granulocyte colony‐stimulating factor receptor. Blood. 1996;88(3):761‐777. 8704229

[6] Nicola NA . Hemopoietic cell growth factors and their receptors. Annu Rev Biochem. 1989;58:45‐77. 2549855 10.1146/annurev.bi.58.070189.000401

[7] Cheers C , Haigh AM , Kelso A , Metcalf D , Stanley ER , Young AM . Production of colony‐stimulating factors (CSFs) during infection: separate determinations of macrophage‐, granulocyte‐, granulocyte‐macrophage‐, and multi‐CSFs. Infect Immun. 1988;56(1):247‐251. 3257205 10.1128/iai.56.1.247-251.1988 PMC259264

[8] Demetri GD , Griffin JD . Granulocyte colony‐stimulating factor and its receptor. Blood. 1991;78(11):2791‐2808. 1720034

[9] Kitagawa S , Yuo A , Souza LM , Saito M , Miura Y , Takaku F . Recombinant human granulocyte colony‐stimulating factor enhances superoxide release in human granulocytes stimulated by the chemotactic peptide. Biochem Biophys Res Comm. 1987;144(3):1143‐1146. 3034272 10.1016/0006-291x(87)91430-6

[10] Yuo A , Kitagawa S , Ohsaka A , et al. Recombinant human granulocyte colony‐stimulating factor as an activator of human granulocytes: potentiation of responses triggered by receptor‐mediated agonists and stimulation of C3bi receptor expression and adherence. Blood. 1989;74(6):2144‐2149. 2553162

[11] Aapro MS , Bohlius J , Cameron DA , et al. 2010 update of EORTC guidelines for the use of granulocyte‐colony stimulating factor to reduce the incidence of chemotherapy‐induced febrile neutropenia in adult patients with lymphoproliferative disorders and solid tumours. Eur J Cancer. 2011;47(1):8‐32. 21095116 10.1016/j.ejca.2010.10.013

[12] Becker PS , Griffiths EA , Alwan LM , et al. NCCN guidelines insights: hematopoietic growth factors, Version 1.2020. Journal of National Comprehensive Cancer. Network. 2020;18(1):12‐22. 10.6004/jnccn.2020.0002 31910384

[13] Zamboni WC . Pharmacokinetics of pegfilgrastim. Pharmacotherapy. 2003;23(8 Pt 2):9S‐14S. 12921217 10.1592/phco.23.9.9s.32888

[14] Crawford J . Safety and efficacy of pegfilgrastim in patients receiving myelosuppressive chemotherapy. Pharmacotherapy. 2003;23(8 Pt 2):15S‐19S. 12921218 10.1592/phco.23.9.15s.32889

[15] Wang Y , Chen L , Liu F , et al. Efficacy and tolerability of granulocyte colony‐stimulating factors in cancer patients after chemotherapy: a systematic review and Bayesian network meta‐analysis. Sci Rep. 2019;9(1):15374. 31653961 10.1038/s41598-019-51982-4 PMC6814815

[16] Botteri E , Krendyukov A , Curigliano G . Comparing granulocyte colony‐stimulating factor filgrastim and pegfilgrastim to its biosimilars in terms of efficacy and safety: A meta‐analysis of randomised clinical trials in breast cancer patients. Eur J Cancer. 2018;89:49‐55. 29227817 10.1016/j.ejca.2017.10.034

[17] Harbeck N , Wang J , Otto GP , Gattu S , Krendyukov A . Safety analysis of proposed pegfilgrastim biosimilar in Phase I and Phase III studies. Future Oncol. 2019;15(12):1313‐1322. 30834780 10.2217/fon-2018-0878

[18] Bellon A , Wang J , Skerjanec A , et al. A large multicentre, randomized, double‐blind, cross‐over study in healthy volunteers to compare pharmacokinetics, pharmacodynamics and safety of a pegfilgrastim biosimilar with its US‐ and EU‐reference biologics. Br J Clin Pharmacol. 2020;86(6):1139‐1149. 32022282 10.1111/bcp.14226 PMC7256126

[19] Kahan Z , Grecea D , Smakal M , et al. Efficacy and safety of RGB‐02, a pegfilgrastim biosimilar to prevent chemotherapy‐induced neutropenia: results of a randomized, double‐blind phase III clinical study vs. reference pegfilgrastim in patients with breast cancer receiving chemotherapy. BMC Cancer. 2019;19(1):122. 30727980 10.1186/s12885-019-5329-6 PMC6364429

[20] Cheson BD , Fisher RI , Barrington SF , et al. Recommendations for initial evaluation, staging, and response assessment of Hodgkin and Non‐Hodgkin lymphoma: the Lugano classification. J Clin Oncol. 2014;32(27):3059‐3067. 25113753 10.1200/JCO.2013.54.8800 PMC4979083

[21] Mehta HM , Malandra M , Corey SJ . G‐CSF and GM‐CSF in neutropenia. J Immunol. 2015;195(4):1341‐1349. 26254266 10.4049/jimmunol.1500861 PMC4741374

[22] Bendall LJ , Bradstock KF . G‐CSF: from granulopoietic stimulant to bone marrow stem cell mobilizing agent. Cytokine Growth Factor Rev. 2014;25(4):355‐367. 25131807 10.1016/j.cytogfr.2014.07.011

[23] Desai K , Misra P , Kher S , Shah N . Clinical confirmation to demonstrate similarity for a biosimilar pegfilgrastim: a 3‐way randomized equivalence study for a proposed biosimilar pegfilgrastim versus US‐licensed and EU‐approved reference products in breast cancer patients receiving myelosuppressive chemotherapy. Exp Hematol Oncol. 2018;7:22. 30202638 10.1186/s40164-018-0114-9 PMC6127997

[24] Gladkov O , Moiseyenko V , Bondarenko IN , et al. Phase II dose‐finding study of balugrastim in breast cancer patients receiving myelosuppressive chemotherapy. Med Oncol. 2015;32(6):623. 25966791 10.1007/s12032-015-0623-x

[25] Harbeck N , Lipatov O , Frolova M , et al. Randomized, double‐blind study comparing proposed biosimilar LA‐EP2006 with reference pegfilgrastim in breast cancer. Future Oncology. 2016;12(11):1359‐1367. 27020170 10.2217/fon-2016-0016 PMC5705792

[26] Avalos BR . The granulocyte colony‐stimulating factor receptor and its role in disorders of granulopoiesis. Leukemia & Lymphoma. 1998;28(3–4):265‐273. 9517498 10.3109/10428199809092682

[27] Larsen A , Davis T , Curtis BM , et al. Expression cloning of a human granulocyte colony‐stimulating factor receptor: a structural mosaic of hematopoietin receptor, immunoglobulin, and fibronectin domains. J Exp Med. 1990;172(6):1559‐1570. 2147944 10.1084/jem.172.6.1559 PMC2188748

[28] Morikawa K , Morikawa S , Nakamura M , Miyawaki T . Characterization of granulocyte colony‐stimulating factor receptor expressed on human lymphocytes. Br J Haematol. 2002;118(1):296‐304. 12100165 10.1046/j.1365-2141.2002.03574.x

[29] Sun R‐F , Yu Q‐Q , Young KH . Critically dysregulated signaling pathways and clinical utility of the pathway biomarkers in lymphoid malignancies. Chronic Dis Transl Med. 2018;4(1):29‐44. 29756121 10.1016/j.cdtm.2018.02.001 PMC5938286

[30] Alas S , Bonavida B . Rituximab inactivates signal transducer and activation of transcription 3 (STAT3) activity in B‐Non‐Hodgkin’s lymphoma through inhibition of the Interleukin 10 autocrine/paracrine loop and results in down‐regulation of Bcl‐2 and sensitization to cytotoxic drugs. Can Res. 2001;61(13):5137‐5144. 11431352

[31] Deng QI , Bai X , Lv HR , Xiao X , Zhao MF , Li YM . Anti‐CD20 antibody induces the improvement of cytokine‐induced killer cell activity via the STAT and MAPK/ERK signaling pathways. Exper Therapeutic Med. 2015;9(4):1215‐1222. 10.3892/etm.2015.2264 PMC4353788 25780412

[32] Dror Y , Ward AC , Touw IP , Freedman MH . Combined corticosteroid/granulocyte colony‐stimulating factor (G‐CSF) therapy in the treatment of severe congenital neutropenia unresponsive to G‐CSF: Activated glucocorticoid receptors synergize with G‐CSF signals. Exp Hematol. 2000;28(12):1381‐1389. 11146160 10.1016/s0301-472x(00)00544-0

[33] Winiarska M , Bojarczuk K , Pyrzynska B , et al. Inhibitors of SRC kinases impair antitumor activity of anti‐CD20 monoclonal antibodies. MAbs. 2014;6(5):1300‐1313. 25517315 10.4161/mabs.32106 PMC4622538

[34] Walsh CA , Qin L , Tien JC‐Y , Young LS , Xu J . The function of steroid receptor coactivator‐1 in normal tissues and cancer. Int J Biol Sci. 2012;8(4):470‐485. 22419892 10.7150/ijbs.4125 PMC3303173

[35] Nakamaki T , Baba Y , Abe M , et al. Rituximab‐induced CD20‐mediated signals and suppression of PI3K‐AKT pathway cooperates to inhibit B‐Cell lymphoma growth by down‐regulation of Myc. Blood. 2016;128(22):5312.

[36] Srinivasan S , Fu G , Dhananjayan S , Zafar S , Nawaz Z . Steroid hormone receptor coactivator, E6‐associted protein (E6‐AP) regulates PI3K‐Akt signaling and prostate cell growth. Can Res. 2007;67(Suppl 9):4152.

